# Extracellular mRNA detected by molecular beacons in tethered lipoplex nanoparticles for diagnosis of human hepatocellular carcinoma

**DOI:** 10.1371/journal.pone.0198552

**Published:** 2018-06-07

**Authors:** Xinmei Wang, Kwang Joo Kwak, Zhaogang Yang, Aili Zhang, Xiaoli Zhang, Rachael Sullivan, Dan Lin, Robert L. Lee, Carlos Castro, Kalpana Ghoshal, Carl Schmidt, L. James Lee

**Affiliations:** 1 Department of Chemical and Biomolecular Engineering, The Ohio State University, Columbus, Ohio, United States of America; 2 College of Pharmacy, The Ohio State University, Columbus, Ohio, United States of America; 3 Center of Biostatistics, The Ohio State University, Columbus, Ohio, United States of America; 4 Department of Surgery and James Cancer Hospital, The Ohio State University, Columbus, Ohio, United States of America; 5 Department of Mechanical Engineering, The Ohio State University, Columbus, Ohio, United States of America; 6 Department of Pathology, The Ohio State University, Columbus, Ohio, United States of America; 7 Comprehensive Cancer Center, The Ohio State University, Columbus, Ohio, United States of America; Kaohsiung Medical University, TAIWAN

## Abstract

Hepatocellular carcinoma (HCC) remains one of the major causes of cancer related deaths. Although ultrasonography (US), computed tomography (CT) and/or high-cost magnetic resonance imaging (MRI) have been shown to improve early detection of liver cancer and mortality rates in high-risk individuals, such imaging based methods are limited by high rates of false positivity leading to unnecessary patient anxiety and invasive procedures. Complementary blood biomarkers could increase the accuracy of early detection. Although Alpha-fetoprotein (AFP) in blood is widely used in HCC screening and diagnosis, the false-negative rate as high as 30% and 40% is found in advanced HCC and early stage HCC respectively. We detected AFP messenger RNA (mRNA) in extracellular vesicles (EVs) in patient plasma using designed molecular beacons and a novel tethered lipoplex nanoparticle (TLN) biochip. Together with glypican-3 (GPC-3) mRNA, another well-known HCC marker, we observed much improved performance of AFP protein-based HCC detection. Comparing normal donors (N = 38) and HCC patients (N = 40), our TLN biochip using EV AFP and GPC-3 mRNAs provided an AUC (area under the ROC curve) of 0.995, better than that of a single marker. This 2-mRNA combination also provided a perfect positive predictive value (PPV = 1) at a negative predictive value (NPV) of 0.95 and 20% prevalence, while the blood AFP protein or plasma EV GPC3 mRNA alone could only provide a PPV of 0.61 and 0.79 respectively at the same conditions. Thus, this facile new method may complement current models for risk stratification in liver cancer screening, therapeutic monitoring, and after-treatment surveillance. However, large scale validation will need to be conducted to confirm its clinical potential.

## Introduction

Hepatocellular carcinoma (HCC) is a high-ranking malignancy with poor treatment outcome. Currently, the diagnosis of HCC relies on radiology imaging including ultrasonography (US), computed tomography (CT), and/or high-cost magnetic resonance imaging (MRI). However, high false-positive rates, high-cost and heavy dependence on the experience of the examiner limit the diagnosis of small malignant lesions by imaging [1]. Although Alpha-fetoprotein (AFP) is widely used in HCC screening and diagnosis, the sensitivity and specificity of AFP protein in HCC patients vary widely with as high as 30% and 40% false-negative rate in advanced and early stage HCCs, respectively [2]. Glypican-3 (GPC-3), a cell-surface protein that is over-expressed in HCC and plays a crucial role in HCC cell proliferation and metastasis [3,4]. It mediates oncogenesis involving signaling pathways during hepatocyte malignant transformation.

Loss-of-function mutations of GPC-3 cause Simpson-Golabi-Behmel syndrome, a rare X-linked overgrowth condition. GPC-3 is a promising candidate for liver cancer therapy given that it shows high expression in HCC, making it a useful biomarker for HCC diagnosis in tumor tissue and cells [3,4]. Recently, the discovery of circulating coding and non-coding RNAs as novel biomarkers in serum or plasma represents a new approach for diagnostic screening, patient prognosis and patient response to therapy because of the non-invasive, objective, and reproducible assessments they can potentially provide [5]. A large number of circulating microRNAs in blood have been investigated in many liver cancer biomarker studies with mixed performance and often inconsistent results without clear understanding of their biological functions [6–10]. Herein, we applied a newly developed technology, tethered lipoplex nanoparticle (TLN) biochip containing molecular beacons (MBs) to simultaneously capture individual EVs and quantify their target mRNAs as potential blood-based biomarkers, which have not been reported in HCC diagnosis [11,12]. We designed specific molecular beacons for AFP and GPC-3 mRNA and tested those using our TLN biochip to evaluate their feasibility as an assay for HCC detection.

## Materials and methods

### Study populations

All the patient EDTA plasma samples were collected from the Ohio State University (OSU) James Cancer Hospital by the authors of this study. 40 HCC-diagnosed patients provided written-informed consent with IRB protocol (Comprehensive Cancer Center (CCC) Clinical Scientific Review Committee (CSRC), Protocol No: #2013C0032). The healthy plasma samples of 38 age-matched healthy individuals were purchased from Zen-Bio, Inc (Research Triangle Park, NC). EDTA Plasma samples from 38 healthy individuals and 40 advanced HCC patients stored at −80°C. All 40 patients were clearly diagnosed as having HCC based on the clinicopathologic findings. The clinicopathological features of the 40 patients are shown in Table 1.

**Table 1:** Clinicopathological characteristics of patients with hepatocellular carcinoma (HCC).

Total No. Percent (%)		
**HCC**	40	100
**Female**	12	30%
**Average Age**	57.5+/-11.5	
**Male**	28	70%
**Average Age**	57.0+/-8.3	
**HBV**	2	5%
**HCV**	27	67.5%
**other**	11	27.5%
**Cirrhosis**	40	100%
**AFP>20ng/ml**	27	67.5%
Healthy donors	**38**	**100%**
**Female**	10	26.3%
**Average Age**	53.5+/-4.1	
**Male**	28	73.7%
**Average Age**	55.0+/-5.8	

6-mL blood samples were collected by veni- puncture or through a Porta-a-Cath implantable venous access system, into a Vacutainer containing potassium EDTA. The blood samples were gently inverted and centri- fuged within 10 minutes at 4°C for 10 minutes. Plasma samples were frozen upright at ≤−70°C within 20 minutes of centrifugation and kept frozen until ready for use.

### Quantitative Reverse Transcriptase PCR (qRT-PCR)

To measure the expression of EV mRNA (AFP and GPC-3) in plasma samples, total EV isolation reagents (Life Technology or SBI ExoQuick, USA) were used to concentrate EVs from plasma and the total RNA in the sample was first transcribed into cDNA by the High Capacity cDNA Reverse Transcription Kit and amplified by qRT-PCR [13]. In detail, frozen plasma samples were thawed on ice until samples were completely liquid and then centrifuged at 2,000 g for 30 min to remove any cellular debris. The supernatant was collected, and 1,00 μL sample was transferred to a fresh tube. Next, each plasma sample was combined with 1/5th volume of Total exosome isolation reagent (SBI, USA) and mixed well by pipetting up and down until a homogenous solution was formed. The samples were incubated at 4°C for 30 min and then centrifuged at 4°C at 1,000 g for 30 min. The supernatant was aspirated and discarded, and the exosome pellet was used for total exosomal RNA extraction. The mirVana RNA isolation kit (Catalog #:AM1561, Life Technology, USA) was utilized for recovery of RNA from the exosome samples obtained with the reagent. 500 μL Lysis/Binding Solution was added to the tube to resuspend the exosome pellet, and vortex vigorously to completely lyse the pellet to obtain a homogenous lysate. 50 μL of Homogenate Additive solution was added to the lysate and the samples were incubated on ice for 10 min. After adding 500 μL Acid-Phenol: Chloroform solution, samples were vortexed vigorously for 60 sec, and centrifuged at 10,000 g for 5 min at room temperature. The aqueous phase was collected and 1.25 volume of room temperature 100% ethanol was added. The mixture was pipetted onto the Filter Carridge, and centrifuged at 10,000 g for 15 sec. Samples were then washed once with 700μL Wash Solution 1 and 2x with 500μL Wash Solution 2/3 (centrifuged at 10,000 g for 15 sec for each wash). After washing, filter was dried by spinning for an additional 1 min at 10,000 g. The filter cartridge was transferred into a fresh collection tube and 30μL of preheated (95°C) nuclease-free water was applied to the center of the filter. Samples were centrifuged for 30 sec at 10,000 g to recover the RNA.

To measure the expression of mRNA (including GAPDH, AFP and GPC-3), the total RNA was first transcribed into cDNA by the High Capacity cDNA Reverse Transcription Kit and further amplified by qRT-PCR. Taqman primers are purchase from Thermofisher, USA. All the results were normalized relative to Cel-miR-39 that of to correct differences in template input.

### Detection of serum AFP by ELISA

For the Alpha-fetoprotein secretion assay, the quantification was performed using an Alpha Fetoprotein Human SimpleStep ELISA kit (Abcam ab193765) according the manufacturer’s instructions. 50 μL of plasma samples or standard were separately added to appropriate wells followed by 50 μL of Antibody Cocktail. The 96-well plate was sealed and incubated for 1 hour at room temperature on a plate shaker set to 400rpm. The plate was washed according to the routine method. Finally, 50 μL of stop solution was added and absorbance was read at 450 nm. The reference value of circulating AFP in healthy subjects was under 20 ng/mL.

### Tethered lipoplex nanoparticle (TLN) assay of plasma samples

**Fig 1:**
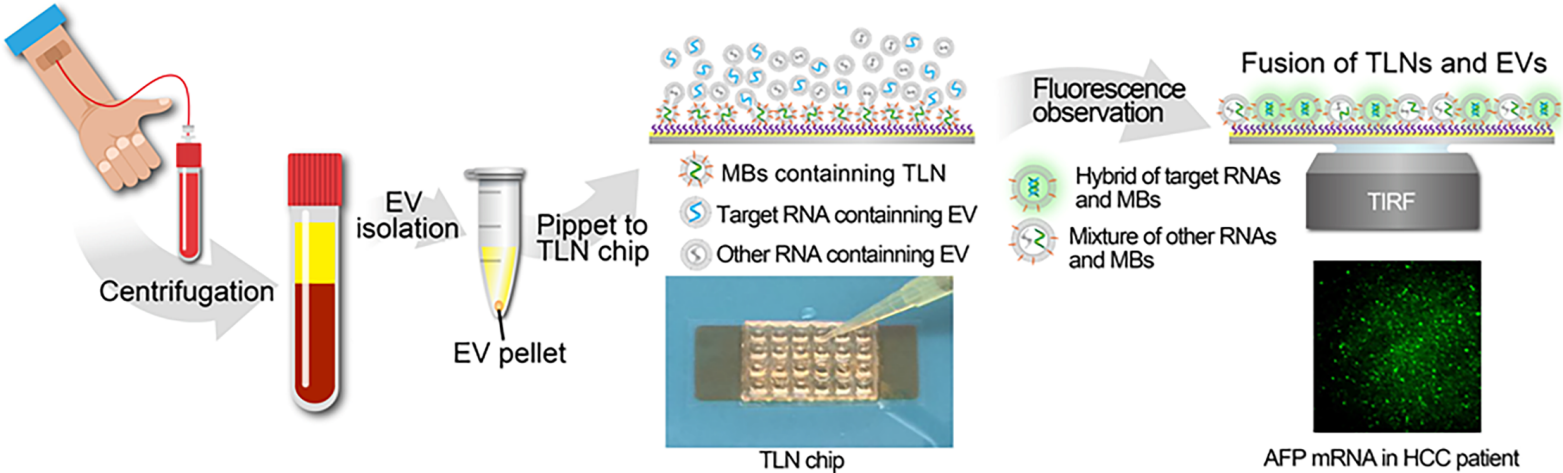
The concept of TLN in EV capture and target mRNA detection, a 24-well TLN biochip and representative TLN-TIRF images (100X).

The tethered lipoplex nanoparticle (TLN) assay captures individual EVs and quantifies their RNA content without tedious sample preparation [3]. **Fig 1** shows the overall concept, a 24-well TLN biochip, and representative TLN-TIRF images.

Extracellular vesicles (EVs) in plasma were purified by an exosome isolation kit and loaded onto the 24-well TLN biochip. AFP and GPC-3 mRNAs in plasma from liver cancer patients were detected using total internal reflective fluorescence (TIRF) microscopy.

MBs for AFP and GPC-3 mRNA were encapsulated in cationic liposomal nanoparticles separately and tethered on a glass slide, which are able to capture negatively charged EVs by electrical static interactions to form a larger nanoscale complex. This lipoplex-EV fusion leads to mixing of RNAs and MBs within the nanoscale confinement near the biochip interface. Total Internal Reflective Fluorescence (TIRF) microscopy is capable of detecting a single biomolecule and it measures signals <300 nm near the interface, which is where the tethered liposomal nanoparticles locate. High sensitivity and near-interface detection make TIRF microscopy a perfect combination with our TLN biochip for detecting the genetic materials in EVs as biomarkers.

### MB design

Molecular beacons that detected AFP and GPC-3 mRNAs were custom synthesized by Sigma-Aldrich (St. Louis, MO). AFP-174: 5’-FAM-CGCGATC-TATGGTAGCCAGGTCAGC-GATCGCG-BHQ1-3’; AFP MB-1096: 5’-Cy5-CGCGATC-TATTCATGAACAAAACTTGCCA-GATCGCG-BBQ-3’, AFP MB-1171: 5’-FAM-CGCGATC-CACTTCTCCAATAACTCCTGGTA-GATCGCG-BHQ1-3’ and GPC3 MB: 5’- 6FAM-CGCGATC-TCTGGACATACTGGATAGAATCAT-GATCGCG-BHQ1-3’.

### Preparation of tethered lipoplex nanoparticles (TLN) containing MBs

9.75 μL of AFP or GPC-3 MB stock solution (100 μM, in PBS) was first mixed with 29.5 μL lipid stock solution (DOTMA: Cholesterol: Biotin-PEG6-SH = 49:49:2, molar ratio, in ethanol). The MB/lipids mixture was then injected into 675 μL PBS and vortexed for 10 s. The solution was further sonicated for 5 min at room temperature, and used immediately.

### Fabrication of TLN biochips

The TLN biochips were fabricated as described before [11]. Briefly, a 15 nm thick Au layer was formed on a glass cover slip over an MPTMS layer as a glue layer using the Denton e-beam evaporator (DV-502A, Moorsetown, NJ). The freshly prepared Au-coated glass was incubated in a mixture ethanol solution of 1-thiahexa(ethyleneoxide) lipidic anchor molecule WC14 [20-tetradecyloxy-3,6,7,12,15,18, 22-heptaoxahexa-tricontane-1-thiol], a lateral spacer β-mercapto-ethanol (βME), and biotin-SH (molar ratio = 30:70:1, in ethanol) overnight. Then, the glass slide was washed three times with absolute ethanol and air dried. The PDMS chip with 24 wells (3 mm in diameter) was fixed to the glass slide, and 20 μL neutravidin solution (100 μg/uL, in ethanol) was applied to each well at room temperature for 15 min. After washing three time with the PBS to remove the unreacted neutravidin, the prepared cationic lipoplex nanoparticles containing MBs were applied to the glass slide. After incubation at room temperature for 15 min, the untethered CLNs were removed by PBS washing.

### Sample preparation and loading

Exosomes are isolated from serum as described before [11]. Briefly, 100 μL serum was mixed well with 25.2 μl ExoQuick exosome isolation solution, and incubated on ice for 30 min. Then the samples were centrifuged at 1000 g for 30 min at 4°C. The pellet was washed wish 1X DPBS and resuspended in 100 μl 1X DPBS. 20 μL samples were loaded onto the TLN biochip and incubated at 37°C for 2 h. On each chip, two wells with 1XDPBS were used as negative control.

### TLN biochip image capture by TIRF microscopy

Total internal reflection fluorescence (TIRF) microscopy was employed to image the samples. Briefly, a 50 mW 488 nm laser and a 100 mW 640 nm laser each at 10% power were used to excite the designed molecular beacons labeled with FAM MBs. The TIRF laser angle was adjusted and fixed to the critical point where the light is reflected at the glass-liquid interface that an evanescent wave of excitation light decays exponentially over 300 nm. Images were recorded by an Andor iXon EMCCD camera with a 100X oil lens, and the exposure time is 200 ms. For each sample, 100 images (10 x 10) were taken in around 2 min for each well on the TLN biochip.

### Image analysis by MATLAB

MATLAB software was used to analyze the images. First, the fluorescence intensities of all 100 images were calculated automatically by MATLAB, and the images with the intensity that are not in the range of Average ± 2*SD were excluded from further analysis. Then, certain cutoff was applied to the images left to remove the background signal. The cutoff threshold was calculated from control group images (PBS group) where the average image intensity was 3,000,000 after the cutoff.

### TLN assay repeating and normalization

We have performed well-to-well on the same chip and chip-to-chip repeatability for many patient samples using AFP and GPC-3 mRNA MBs. The results showed that the reproducibility was very high and there was little well-to-well difference on the same chip. One or two healthy donors were used to diminish the chip-to-chip variation. After PBS normalization, the values of the positive controls were compared between different chips, and the ratio of the same positive control was calculated and applied for chip-to-chip normalization. All the samples were repeated at least twice in two different chips, and the values were calculated independently. A third assay was performed to those samples if the difference was greater than 20% in two different chips. All the data were averaged and reported as mean ± SD.

### TLN assay sensitivity

TIRF microscopy can detect fluorescence at the single molecule lever. We have demonstrated that our TLN-TIRF assay is able to capture and identify a single extracellular vesicle (EV) or exosome (data not shown). To verify the applicability of our new TLN-TIRF technology, we first collected EVs from culture medium of two well-known cell lines, A549 and HBEC by ultracentrifugation, and then applied to qRT-PCR and the TLN biochip containing both miR-21-specific and TTF1 mRNA-specific molecular beacons (MBs). As reported in our previous publication [11], TLN and qRT-PCR provided comparable results for miR-21 detection. TTF1 mRNA in the EVs was not detected by qRT-PCR, but was clearly detected using the TLN biochip. Even though mRNAs have been routinely detected in tissues and cells by qRT-PCR, their detection in EVs turned out to be much more difficult by qRT-PCR. This is because, unlike in tissue and cells, mRNAs are presented in EVs as a mixture of intact large molecules and smaller fragments. Since qRT-PCR assay is designed to replicate a relatively large portion of mRNA exons (i.e. 100~150 nts), it is not able to detect the presence of small fragments of mRNA targets. Molecular beacon (MB), on the other hand, only hybridizes with 20~30 nts of a specific mRNA exon, so it may detect both intact as well as fragments (large and small) of mRNA targets in EVs. This is why our TLN biochip may provide a much higher sensitivity than qRT-PCR with a much smaller sample size (i.e. <20 vs. ~1,000 μL of patient plasma).

### TLN assay specificity

We performed the specificity experiments using TLN biochips containing miR-21 MB to detect artificial exosomes containing miR-21 and its mismatch mutants. As shown in **S2 Fig**, for the single mutation miR-21, the fluorescence signal was around 60–70% compared to WT miR-21. For the bi-mutation, the signal decreased sharply to <20% of that of the WT, and the tri-mutation signal was <10%. High specificity was achieved by designing the MB to hybridize a sequence which is unique for the miRNA or mRNA target, i.e. without single mismatch with any other microRNAs or mRNAs.

### Dynamic range of TLN assay

In our current TLN assay, we took 100 TIRF images with each image covering an area of 80 μm x 80 μm. The estimated EVs captured were ~10^10^. There are 10^12^~10^14^ EVs in 1 mL blood. More EVs can be captured and detected by our TLN biochip by taking more TIRF images, however, 100 images per sample provide the best balance of accuracy and assay speed [11].

### Statistical analysis

The data was first log2 transformed to reduce skewness and variance. Logistic regression was then used to test whether the individual factor (AFP protein, AFP mRNA, or GPC mRNA) or combination of the two factors (AFP protein+ AFP mRNA, or GPCmRNA + AFP mRNA, or GPCmRNA + AFP protein) are significantly correlated with the odds of having liver tumor. The results showed that each of the individual factors is significantly correlated with the odds of having tumor. With the increase of the mRNA or protein, there is increased risk of being a liver cancer patient; however, for the two factor combination models, the model for GPCmRNA + AFP protein cannot be converged. For the other two combination models, one of the factors (GPCmRNA + AFP mRNA, where AFP mRNA is not significant) (AFP protein+ AFP mRNA, where AFP protein is not significant) is not significantly correlated with the risk of having cancer. Details were described in S3 **Fig** and S1 Table.

## Results

**Fig 2:**
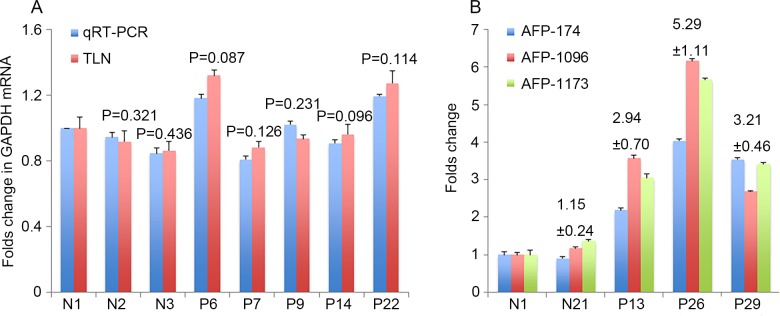
Comparison of qRT-PCR and newly developed TLN assay. (A) Comparable qRT-PCR (200 μl plasma) and TLN (20 μl plasma) assay results on GAPDH levels in plasma EVs. (B) Results of different AFP molecular beacons detecting different locations of AFP mRNA fragments and no significant difference was found among different AFP molecular beacons (P>0.5).

Although mRNAs have been routinely detected in tissue and cells by qRT-PCR, their detection in circulating EVs has been more challenging since mRNAs present in EVs are a mixture of intact and fragmented transcripts [8,9]. The qRT-PCR assay is designed to amplify and detect a larger portion of the transcripts (usually 100~150 nts) and requires at least two sites for PCR primer recognition, so the presence of smaller fragmented transcripts cannot be detected. Molecular beacons (MBs), on the other hand, hybridize to 18~25 nts of a specific mRNA, so it may detect both intact, larger and smaller fragments of mRNA targets in EVs with only 20 μL of plasma as shown in **Fig 1**. For more abundant mRNAs such as GAPDH, both qRT-PCR (Ct values in 28~32 for EVs in 200 μL plasma sample size) and our TLN biochip (EVs in 20 μL plasma sample size) were able to provide very similar results for 3 healthy individuals and 5 HCC patients as shown in **Fig 2A**.

**Fig 3:**
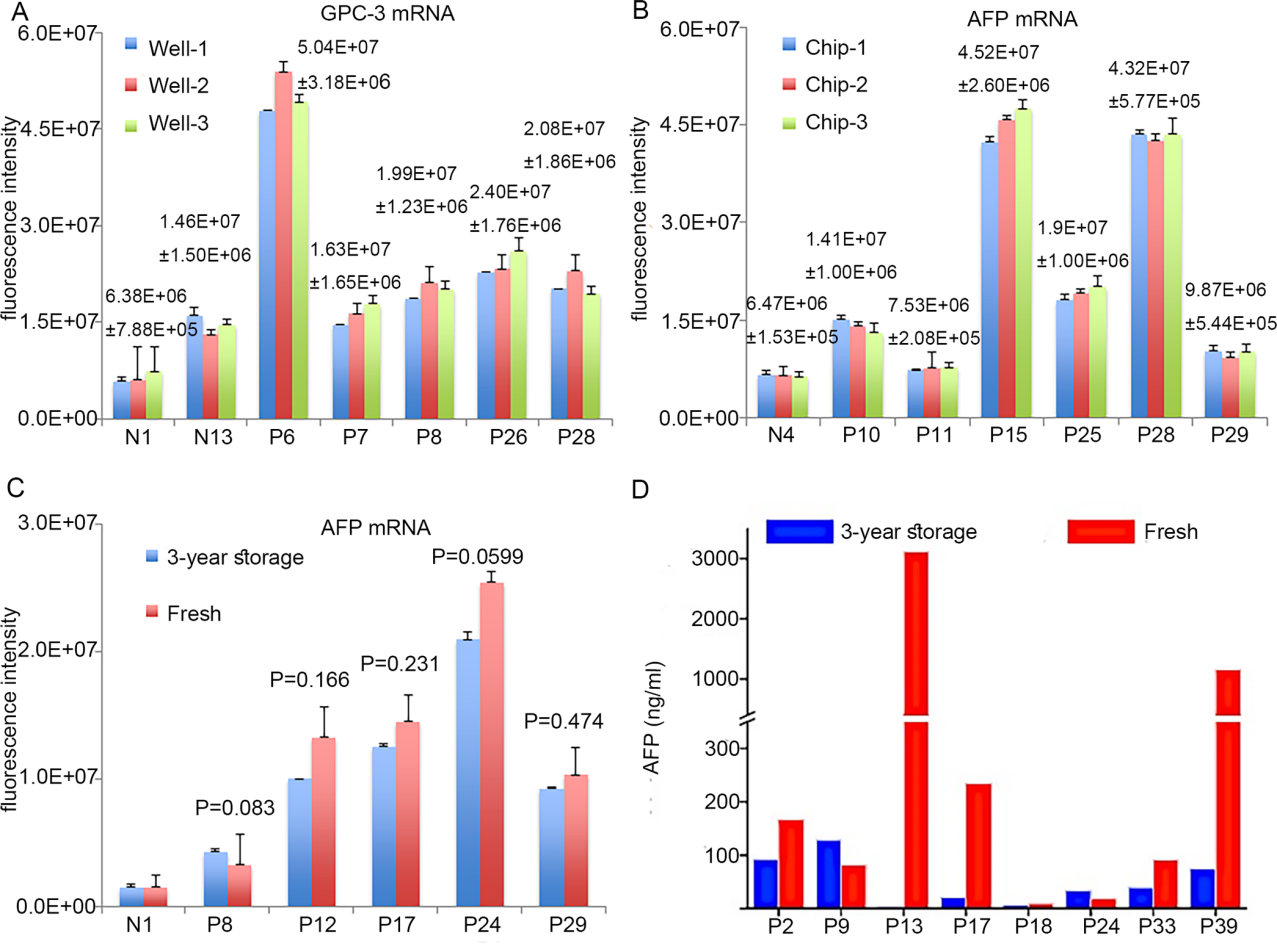
Analysis of repeatability. (A) Repeatability of well-to-well and (B) chip-to-chip in TLN assay. (C) EV-mRNA and (D) protein stability comparison between fresh blood samples and samples after 3-year storage. No significant difference (P>0.5) was observed in the repeating experiments.

The Ct values are indicated in **S1 Fig**. However, both EV AFP and GPC3 mRNAs were ‘undetermined’ in the qRT-PCR assay with 200 μL plasma, while MBs in TLN biochips could detect both EV mRNAs with only 20 μL plasma. In order to confirm that the 20~30 nts on the mRNA fragments detected by MBs can indeed represent the entire mRNA content in all EVs in the plasma sample, three different MBs were designed to target three locations of the ~1200 bp total AFP mRNA, i.e.174-191 (gctgacctggctaccata), 1096–1117 (tggcaagttttgttcatgaata) and 1171–1195 (gataccaggagttattggagaagtg). **Fig 2B** shows that all three MBs could provide comparable EV AFP mRNA expressions in 2 healthy individuals and 3 HCC patients. Although the fold changes varied somewhat among the three MBs because mRNA fragments in EVs would not be exactly the same, the trend and the difference between healthy and HCC samples remained very similar. We selected the AFP-1096 MB as recommended by the provider (Sigma). As shown in **Fig 3A and 3B**, both the well-to-well technical repeatability on the same chip and the chip-to-chip technical repeatability revealed very small variation, 6–10% for 7 samples when 100 μL of plasma was used for EV isolation. Unlike AFP protein, no significant differences in EV AFP mRNA expression were found between fresh blood samples and samples stored for three-years, which suggests that mRNAs in EVs are much more stable than plasma proteins (**Fig 3C and 3D**).

**Fig 4:**
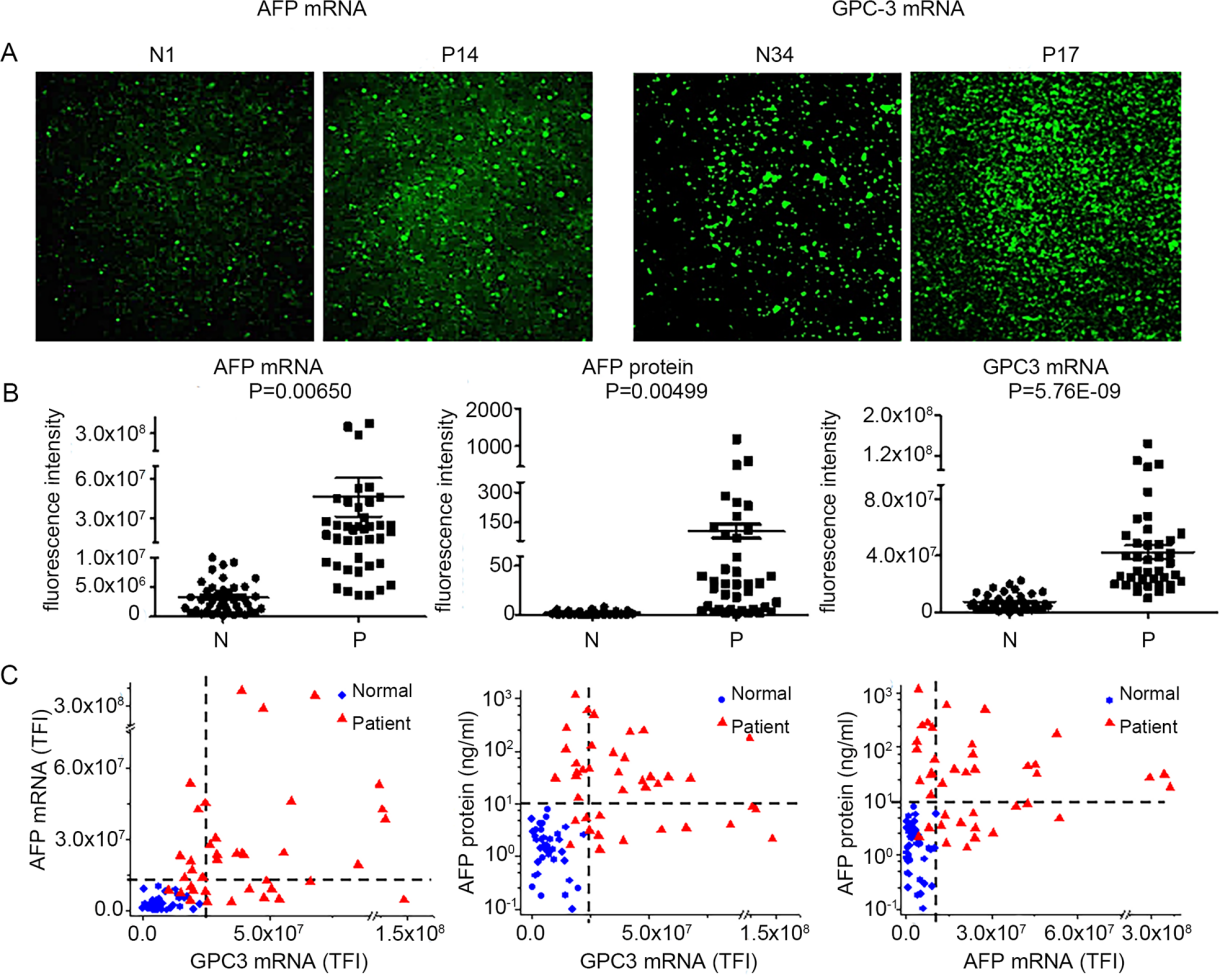
Median expression levels of EV AFP and GPC-3 mRNA are significantly higher in HCC patients than in healthy controls. (A) Representative TLN-TIRF images (80 × 80 μm) (100X) of EV AFP and GPC-3 mRNA expression in a HCC patient and a healthy donor plasma samples. (B) Dot charts of EV AFP and GPC-3 mRNA expression in HCC patient and healthy donor plasma samples. (C) Scatter plots of plasma AFP protein vs. EV GPC-3 mRNA, EV AFP mRNA vs. EV GPC-3 mRNA, and plasma AFP protein vs. EV GPC-3 mRNA between HCC patients and healthy donors.

A total of 78 human plasma samples from 40 HCC patients and 38 normal donors were tested in this study. There are no significant differences of age and sex between the two cohorts. **Fig 4A–4C** show representative Total Internal Reflection Fluorescence (TIRF) microscopy images of TLN biochips, bar charts and scatter plots, respectively of EV AFP and GPC-3 mRNA expressions as well as plasma AFP protein levels for both cohorts. The data shown in **Fig 4B** indicate that the median expression levels of EV AFP and GPC-3 mRNA are significantly higher in HCC patients than in healthy individuals (P = 0.0065 and 5.76E-09 respectively). A similar trend is observed for the expression of plasma AFP protein (P = 0.005). However, there is substantial overlap between the two cohorts using any of the individual marker. The scatter plots in **Fig 4C** show that a combination of any two markers could provide a much better distinction between patients from normal donors. Statistical analysis further shows such a trend. Comparing normal donors and HCC patients, our TLN biochip using EV AFP and GPC-3 mRNAs provided an AUC (area under the ROC curve) of 0.995, better than that of a single marker (0.936 for AFP protein, 0.947 for EV AFP mRNA, and 0.979 for EV GPC3 mRNA). This 2-mRNA combination also provided a perfect positive predictive value (PPV = 1) at a negative predictive value (NPV) of 0.95 and 20% prevalence, while the blood AFP protein or plasma EV GPC3 mRNA alone could only provide a PPV of 0.61 and 0.79 respectively at the same conditions (S1 Table).

## Discussion

Given the high false positive rates inherent in imaging-based liver cancer screening and the high false negative rate of blood AFP protein-based liver cancer detection, it is essential to develop accurate, affordable and minimally invasive assays. Blood-based molecular biomarkers hold promise and have gained a great deal of attention in recent years. Most studies today have focused on circulating microRNAs [14–20] or proteins [2–4] in total RNAs or proteins gathered from the entire serum/plasma sample. The findings usually lack sufficient sensitivity for early stage detection and reproducibility in different patient cohorts [2]. Using the unique TLN biochips and molecular beacons, we have demonstrated the superior performance of EV GPC-3 and AFP mRNA to the conventional AFP protein marker for HCC diagnosis. Our TLN-TIRF assay is also much more sensitive than the conventional qRT-PCR assay for circulating mRNA detection. Larger-scale validation studies at multiple sites must be performed to further support a conclusion that EV containing mRNA targets in blood detected by the new TLN biochip can serve as a viable screening marker to distinguish non-cancer from cancer. Also, other mRNA, microRNA and/or lncRNA targets identified by RNA profiling should be considered to enhance the HCC diagnosis performance, particularly for high-risk populations such as patients with HBV, HCV, cirrhosis, and benign liver nodules because various causes may lead to HCC. Although this new method is promising, it will need more patient samples and detailed clinic characteristics to make the method clinically viable.

## Supporting information

S1 Fig Ct values of GAPDH in HCC patient and healthy donor plasma. (TIF)

S2 Fig TLN specificity tested by miR-21 mutants. (A) Representative TLN-TIRF images (80 μm x 80 μm) for different miR-21 mutants. (B) Fluorescence intensities of different miR-21 mutants measured by Metlab software.
(TIF)

S3 Fig Comparision of ROC curves of individual markers and combined markers. (TIFF)

S1 Table The sensitivity, specificity, and PPV were obtained for each model when NPV is at around 0.95. (DOCX)
