# Supplementary figures and images for: Extracellular mRNA detected by molecular beacons in tethered lipoplex nanoparticles for diagnosis of human hepatocellular carcinoma
Source: PLoS One. 2018 Jun 7;13(6):e0198552. doi: 10.1371/journal.pone.0198552 (PMC5991670; doi:10.1371/journal.pone.0198552)

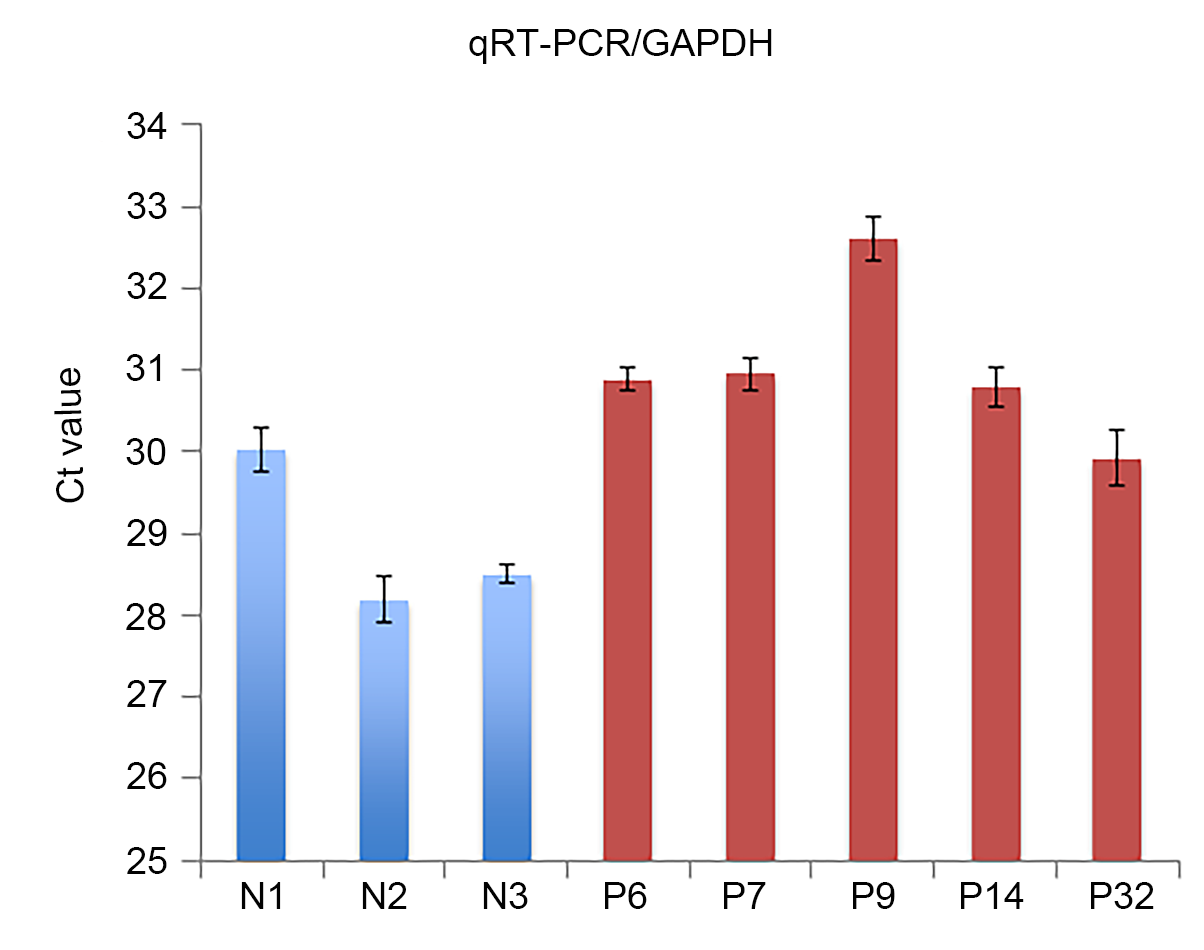

Supplement: S1 Fig — (TIF) [file pone.0198552.s001.tif]

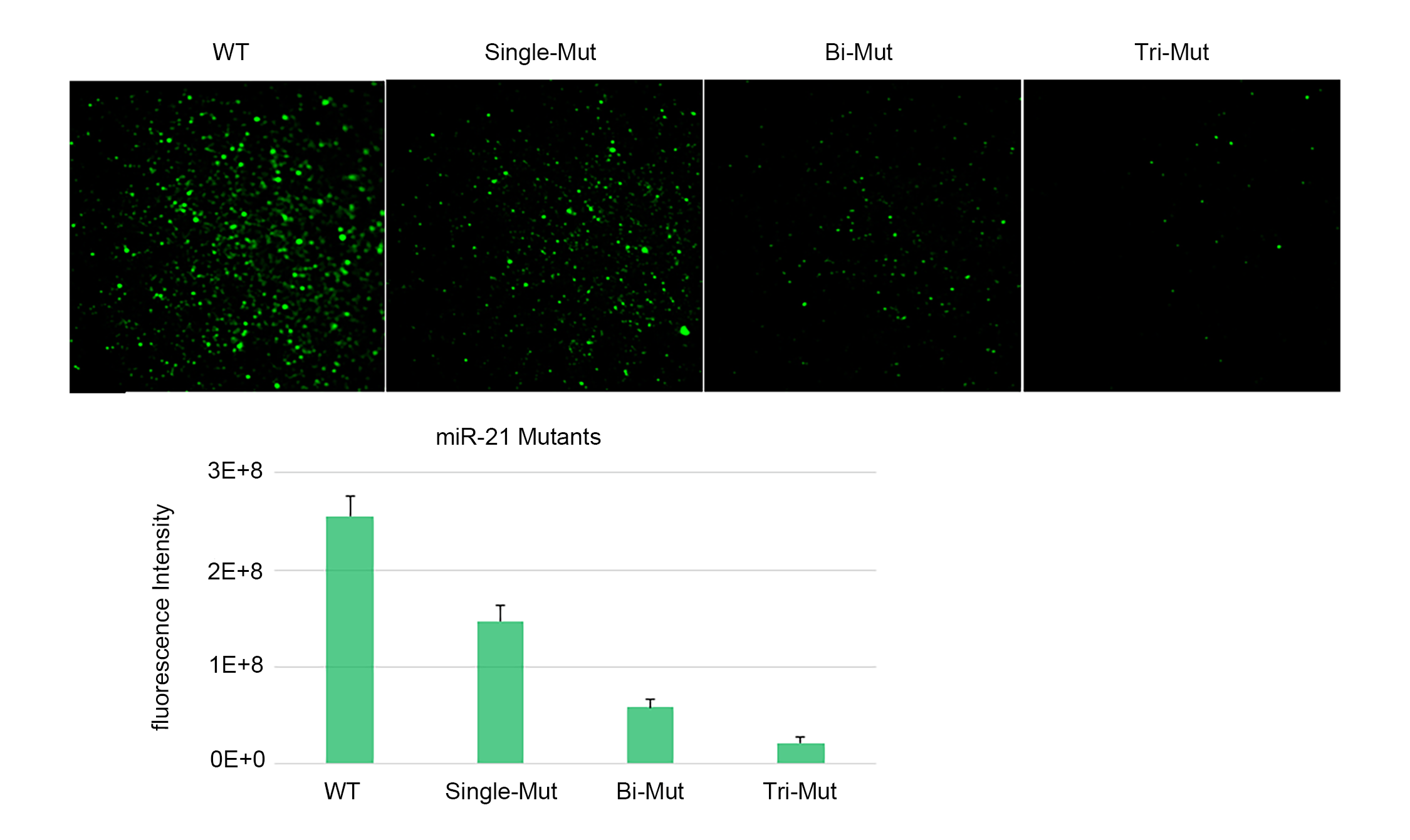

Supplement: S2 Fig — (A) Representative TLN-TIRF images (80 μm x 80 μm) for different miR-21 mutants. (B) Fluorescence intensities of different miR-21 mutants measured by Metlab software. (TIF) [file pone.0198552.s002.tif]

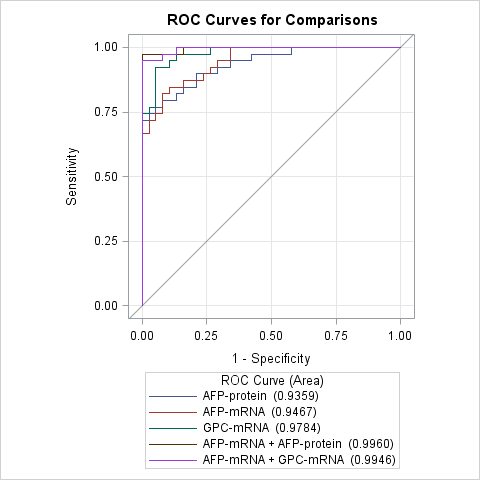

Supplement: S3 Fig — (TIFF) [file pone.0198552.s003.tiff]

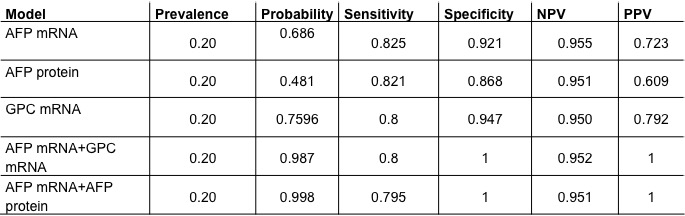
**S1 Table.** The sensitivity, specificity, and PPV were obtained for each model when NPV is at around 0.95.

Supplement: S1 Table — (DOCX) [file pone.0198552.s004.docx]
